# The challenges of introducing a malaria vaccine 

**DOI:** 10.2471/BLT.16.030916

**Published:** 2016-09-01

## Abstract

For the first time a malaria vaccine is to be tested for possible inclusion in national immunization programmes. Malcolm Molyneux tells Fiona Fleck why governments may need to work hard to convince people of its benefits.

Q: How did you become interested in malaria research?

A: I was born in a malarious area in what is now the Democratic Republic of Congo, where my parents were missionaries. I remember as a child frequently hearing a high-pitched noise in my ears. Later I realised it was the effect of the quinine I was given to prevent bouts of malaria. From the age of 13 I lived in the United Kingdom, eventually studying medicine, but I always wanted to get in touch with my childhood environment. Fortunately my wife Liz, a paediatrician who had spent her first 12 years in India, was also keen to travel, and we went to work in Malawi from 1974 until 1984. Later at the Liverpool School of Tropical Medicine I met Herbert Gilles, who inspired me with his enthusiasm for academic tropical medicine and for malaria research in particular.

Q: What was it like treating malaria in those years?

A: When I first arrived in Malawi, I was struck by the mildness of malaria in adults, while its effects were devastating in children. Antimalarial drugs were much misused in patients of all ages, as in the case of a man with an uncomplicated fever who was given a large dose of chloroquine by intramuscular injection. He got into his car and drove straight into a tree, having – presumably – suffered a profound drop in blood pressure: he should have been treated with tablets, and probably didn’t have malaria anyway.

“Antimalarial drugs were much misused in patients of all ages.”

Q. What has your subsequent work in malaria involved?

A: Much of my research at the medical school in Malawi was in collaboration with Terrie Taylor and Malawian colleagues on the characteristics and management of severe malaria in children. One of the things we noticed was that the case fatality for children coming in to the hospital with life-threatening malaria was 20–30%, even with optimal treatment. Children often became ill so rapidly that their parents didn’t have time to administer treatment. If you can treat malaria promptly, the risk of severe disease is reduced, but sometimes there is not enough time for that correct early response. Clearly preventive measures were crucial, and existing methods of prevention, although helpful, were not sufficient; an effective vaccine could have a major impact.

Q: How have you been involved in the development of malaria vaccines?

A: My first experience was when I was asked to be on the data safety monitoring board (DSMB) for a candidate malaria vaccine, developed in Colombia, called SPf66. When the news of the vaccine hit headlines around the world as an end to the curse of malaria in 1993, I went as the chair of the DSMB to [the United Republic of] Tanzania to see how a big trial was being carried out. The results were in the direction of efficacy but with borderline significance. I similarly observed subsequent trials of the same product conducted in Gambia and Thailand, which showed no efficacy at all against malaria infection or disease. Much was learned about how to conduct and interpret trials against malaria, but we still had no vaccine.

Q: What is your involvement with the new vaccine, RTS,S?

A: Phase I trials in the United States of America (USA), reported in 1995, showed that a precursor to the current vaccine could prevent malaria in non-immune volunteers artificially inoculated with *Plasmodium falciparum* parasites. Many trials have since been conducted in populations in malaria-endemic countries, for which I again chaired the DSMB (now called the Independent Data Monitoring Committee). We keep a close eye on what happens to populations exposed to the vaccine, looking for dangers as well as immune responses and benefit against malaria.

Q: This is the first malaria vaccine to complete phase III clinical trials. What is different about RTS,S from previous malaria vaccines?

A: Unlike SPf66, all trials with RTS,S (there have been dozens) have shown efficacy, and usually of about the same degree – around 30–50% reduction of clinical malaria during the following year in children who received the vaccine compared to children in the same areas who did not.

Q: In 2015, there were an estimated 438 000 deaths from malaria and about 78% of these were in children younger than five years. Will the vaccine prevent these deaths? Why did the clinical trials only focus on whether the vaccine reduces disease rather than on whether it reduces deaths? 

A: Deaths from malaria, although a major problem at a national level, are much less frequent than simple malaria fevers. It would take an impossibly large trial to be able to detect a beneficial effect of the vaccine on malaria deaths at this stage of the programme. Severe malaria is more common than fatal malaria and served as a surrogate of malaria mortality in the phase III study. The use of severe disease events as an indicator of likely fatal events assumes that a vaccine that prevents severe malaria would also prevent deaths from malaria, which is a reasonable, if unproven, assumption.

Q: What does the new vaccine RTS,S actually do?

A: When a female *Anopheles *mosquito drinks your blood, she introduces saliva containing parasites called sporozoites into your blood stream. The vaccine includes parts of the sporozoite’s surface coat and immunity induced by the vaccine can impair the capacity of these parasites to complete their development in the liver, and renders them less capable of multiplying in the blood.

Q: Were you disappointed when the results of phase III trials showed that the new vaccine confers only partial protection?

A: No, we had been monitoring smaller trials earlier and we expected such results. I am disappointed that the effect of the vaccine does not last very long and that three doses confer protection for only about a year. But it is encouraging that a fourth dose extends the efficacy for another year or so. By this time a child may have developed his or her own partial immunity and be less susceptible to malaria.

Q: The new malaria vaccine is against the type of malaria that is most common in Africa and mainly affects children, P. falciparum. National immunization programmes in African countries would need to add new vaccination visits to deliver this malaria vaccine. How could immunization programmes accomplish this?

A: The idea is to link these vaccinations to other contacts between the health system and children. Unfortunately, when RTS,S was given at the times of the existing three doses of most vaccines in the expanded programme on immunization (EPI) at 6, 10 and 14 weeks, the efficacy was less than in slightly older children, so at present the World Health Organization (WHO) recommends that children should have the three doses of RTS,S at monthly intervals starting as soon as possible after five months of age. Where feasible the doses could be linked with another activity requiring the child’s attendance at a health facility, such as nutritional assessment, vitamin  A delivery or measles vaccine. We would also need to find an opportunity about 18 months later to deliver the fourth dose.

Q: How realistic is this schedule and the recommendation that the vaccine should be delivered in combination with other measures?

A: We have good evidence that our best chance of controlling the disease is to apply a combination of measures. One of the impressive results of phase III trials of RTS,S is that they were done in areas where other measures against malaria were being used as fully as possible, in particular regular use of bednets and access to prompt treatment of fever: it was against that background that the RTS,S vaccine produced about 40% efficacy. Habits can be changed by circumstances. People can be persuaded to take preventive measures especially against a threat to their children’s lives.

“People can be persuaded to take preventive measures especially against a threat to their children’s lives.”

Q: How can countries afford this? Wouldn’t it be better to focus on fewer interventions that are known to be effective?

A: There are precedents: HIV and tuberculosis treatment programmes have been funded by international institutions. GAVI may support RTS,S vaccination since its board has approved funding for the malaria pilot implementation programme. Vaccination is one of the most efficient kinds of intervention against disease. With few contacts with health workers and no other behavioural changes required, considerable benefits can be achieved; these are important considerations when assessing the new vaccine’s cost–effectiveness.

Q: What conditions are required to make the inclusion of the new vaccine into national immunization programmes in endemic countries in Africa a success?

A: The vaccine needs to be recommended by WHO; currently it isn't. The vaccine must first be evaluated in subnational pilot implementation projects as recommended by WHO. Once these hurdles are clear, as for introducing any new vaccine, there needs to be a sufficiently distributed health infrastructure, with sufficient staff at all levels who have the knowledge, enthusiasm and facilities to provide it.

Q: The new vaccine does not show high efficacy in trials, and efficacy may be even lower when delivered through national programmes. Could inclusion of the new vaccine lead to perceptions that vaccines are not effective?

A: Pneumococcal vaccines don’t protect against all pneumonia, and rotavirus vaccines don’t protect against all diarrhoea, so it may not be so much of a problem. Still, a vaccine that is partially efficacious is a difficult concept to grasp. We need to raise public awareness of the threat that malaria poses to the lives of children, and the benefit of the new vaccine in combination with other interventions. 

